# Digital health meets Parkinson’s disease: a bibliometric exploration of wearable sensors research and emerging frontiers (2005–2026)

**DOI:** 10.3389/fninf.2026.1937585

**Published:** 2026-10-08

**Authors:** Lan Xiong

**Affiliations:** Department of Neurology, The First People's Hospital of Guangyuan, Guangyuan, Sichuan, China

**Keywords:** artificial intelligence, bibliometric analysis, digital biomarkers, Parkinson’s disease, research trends, wearable and sensor-based monitoring technologies

## Abstract

**Background:**

Wearable and sensor-based monitoring technologies are increasingly used to support objective, longitudinal assessment in Parkinson’s disease (PD). However, the global development, knowledge structure, and emerging research frontiers of this interdisciplinary field remain incompletely mapped.

**Methods:**

Bibliographic records on wearable and sensor-based monitoring technologies in PD were retrieved from the Web of Science Core Collection (WoSCC) and Scopus for the period from 1 January 2005 to 2 February 2026. Searches were conducted in title, abstract, and author keyword fields. Microsoft Excel, VOSviewer, CiteSpace, and the bibliometrix R package were used to analyze publication trends, collaboration networks, co-citation patterns, keyword co-occurrence, clustering, citation bursts, historiographic patterns, and source impact.

**Results:**

A total of 3,332 publications were included. Publication output increased rapidly over the study period, particularly after 2019, while 2026 represented an incomplete publication year because the search was conducted on 2 February 2026. The United States (U.S.), China, and European countries constituted the main knowledge-production and collaboration structure. Thematic analyses suggested a gradual shift from engineering validation and objective motor-symptom quantification toward AI-enabled digital biomarkers, remote monitoring, multimodal monitoring, and closed-loop neuromodulation. These patterns reflect research activity and citation structures rather than direct evidence of clinical efficacy.

**Conclusion:**

This bibliometric analysis provides science-mapping evidence on the evolution of wearable and sensor-based monitoring technologies in PD. Future work should prioritize standardization, multicenter real-world validation, clinical workflow integration, multimodal data fusion, and cautious evaluation of closed-loop applications.

## 1 Introduction

Parkinson’s disease (PD) is a chronic, progressive neurodegenerative disorder characterized by substantial temporal variability in motor and non-motor symptoms, treatment responses, and functional status. Conventional outpatient assessments, including the Movement Disorder Society-sponsored revision of the Unified Parkinson’s Disease Rating Scale (MDS-UPDRS), remain essential but are episodic, clinic-based, and partly subjective, limiting their ability to capture patients’ real-world symptom fluctuations and functional status (Espay et al., 2016; Goetz et al., 2008). This gap has driven increasing interest in repeated or continuous monitoring technologies that can capture clinically relevant changes in gait, tremor, bradykinesia, dyskinesia, sleep, and daily activity in real-world settings.

Wearable and sensor-based monitoring technologies have emerged as practical tools for capturing objective, longitudinal, and ecologically relevant information in PD (Maetzler et al., 2013). These approaches encompass wearable, physiologic, ambulatory, and remote monitoring technologies and biosignal monitoring approaches (Bougea, 2024; Majumder et al., 2017). By capturing movement, physiological signals, and real-world activity, these technologies may quantify symptom variability and complement clinical assessment (Burq et al., 2022; Espay et al., 2019). Advances in artificial intelligence (AI) and machine learning have further enabled automated analysis of sensor-derived data for PD assessment (di Biase et al., 2024; Gorce and Jacquier-Bret, 2026). Accordingly, research in this field has expanded across a broad range of clinical domains, including motor symptoms, functional performance, freezing of gait (FOG), falls, and treatment-related outcomes (Bougea, 2025; Huang et al., 2023; Qi et al., 2025; Rovini et al., 2017). Despite this rapid expansion and increasing interdisciplinary integration, the review literature has largely developed along parallel, topic-specific lines. Existing reviews have addressed distinct aspects of wearable and sensor-based research in PD, including wearable technologies and monitoring applications (Rovini et al., 2017, 2019), specific motor manifestations such as FOG and falls (Huang et al., 2023; Silva de Lima et al., 2017), digital biomarkers for non-motor symptoms (Janssen Daalen et al., 2024), and machine learning-assisted diagnosis (di Biase et al., 2024). While previous reviews offer valuable insights into specific niches, they often lack a macro-level perspective on the field’s evolution. Written primarily for a clinical and translational readership—including movement disorder specialists and digital health practitioners—this analysis focuses on the clinical deployment of sensor technologies. To serve this audience, it is crucial to understand how these applications have diversified across patient-facing versus clinician-facing interfaces, early to advanced PD stages, and the tracking of both motor and non-motor symptoms. Furthermore, their intended clinical purposes have rapidly expanded, ranging from early diagnostic support and objective severity assessment to continuous home monitoring and patient self-management. Given this highly complex clinical landscape, the overarching intellectual structure, collaborative networks, and thematic trajectories of this field remain insufficiently mapped.

Bibliometric analysis can complement conventional reviews by mapping the development, knowledge structure, and research trends of a field. The present bibliometric study aimed to map the global research landscape of wearable and sensor-based monitoring technologies in Parkinson’s disease from 2005 to 2 February 2026. We analyzed publication trends, influential countries, institutions, authors, and journals, co-citation, keyword co-occurrence, thematic evolution, citation bursts, and historiographic patterns. The objective was to describe global trends, identify the field’s knowledge foundations, trace thematic evolution, and highlight emerging frontiers for future research and clinical translation.

## 2 Methods

### 2.1 Database and search strategy

To construct our search query, we combined two primary conceptual domains: Parkinson’s disease terminology and wearable monitoring technologies. The specific keywords and synonyms populating these domains were extracted from key literature and joined using Boolean operators. While the underlying logic remained identical, the final search strings were formatted to accommodate the specific syntax constraints of WoSCC and Scopus (see Supplementary Table 4).

We validated this approach prior to data extraction. A benchmark cohort of eight highly relevant papers—covering domains such as continuous ambulatory tracking, freezing-of-gait detection, and digital smartphone assessments—was selected from our initial scoping review. We tested our finalized query against this cohort to assess its sensitivity. The search successfully retrieved seven out of the eight benchmark texts (87.5%) across both databases, with a single article remaining unindexed under these parameters. Supplementary Table 5 details the precise search codes and corresponding validation outcomes.

Only English-language articles and review articles were included. The initial search yielded 1,443 records from WoSCC and 3,322 records from Scopus. After merging records from the two databases, removing duplicates, and excluding records outside the study scope or with incomplete essential bibliographic information, 3,332 publications were retained for bibliometric analysis.

### 2.2 Data processing

All retrieved records were exported with full bibliographic information and cited references where available. Microsoft Excel 2021 was used for data organization, deduplication checks, descriptive summaries, and annual publication trend analysis, including annual and cumulative publication output.

VOSviewer version 1.6.20 was used to construct and visualize bibliometric networks, including co-authorship networks, co-citation networks, and keyword co-occurrence networks (van Eck and Waltman, 2010). CiteSpace version 6.4.R1 was used for clustering analysis, timeline visualization, centrality assessment, and citation burst detection to identify influential references, emerging topics, and temporal changes in the field (Chen et al., 2014; Synnestvedt et al., 2005). Citation burst detection was interpreted as a marked increase in citation frequency within a defined period; burst strength indicated the intensity of the increase, and start and end years indicated its duration.

The bibliometrix R package was used for complementary bibliometric analyses, including source impact assessment, historiographic analysis, and the calculation of indicators such as the h-index, g-index, number of citations (NC), and number of publications (NP) (Aria and Cuccurullo, 2017). Together, these tools allowed separate assessment of publication trends, co-authorship, co-citation, keyword co-occurrence, clustering, burst detection, and historiographic/source impact (Figure 1).

**Figure 1:**
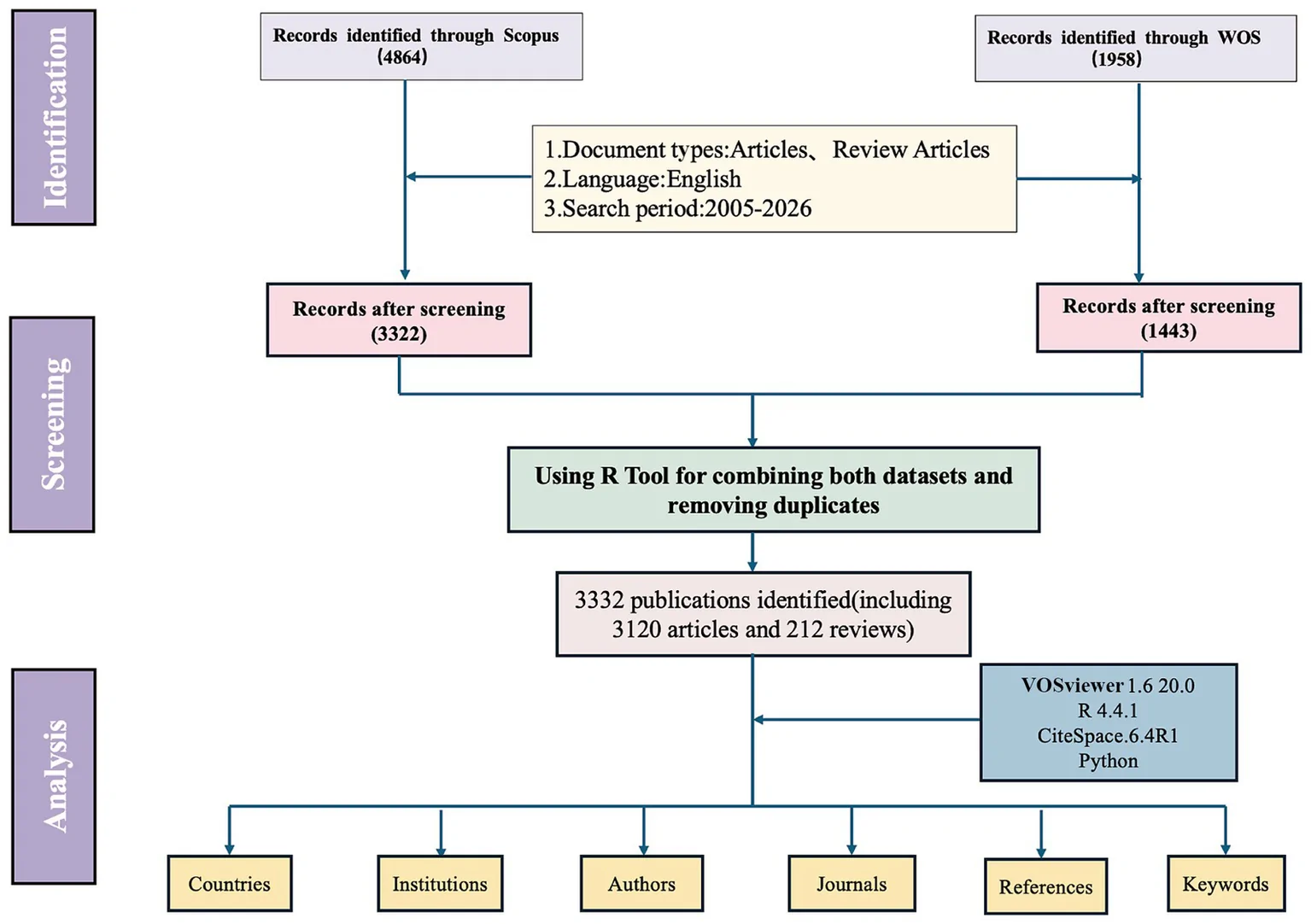
The flowchart for literature search, selection and analysis.

## 3 Results

### 3.1 Publication trends and author networks

A total of 3,332 documents published from 2005 to 2 February 2026 were included (with 2026 representing an incomplete year). Original articles dominated the dataset (93.64%), indicating a field driven heavily by primary experimental research rather than reviews (Figure 2A). Annual publication output grew exponentially, remaining below 100 before 2019 but exceeding 700 by 2025 (Figure 2B). This trajectory fits an exponential growth model (y = 2.9369e0.3436x, *R*^2^ = 0.9442), reflecting a rapid and sustained acceleration in research interest, likely driven by recent advancements in AI and remote care. Detailed bibliometric metadata, including keyword distributions and total citation volumes, are summarized in Supplementary Table 1.

**Figure 2:**
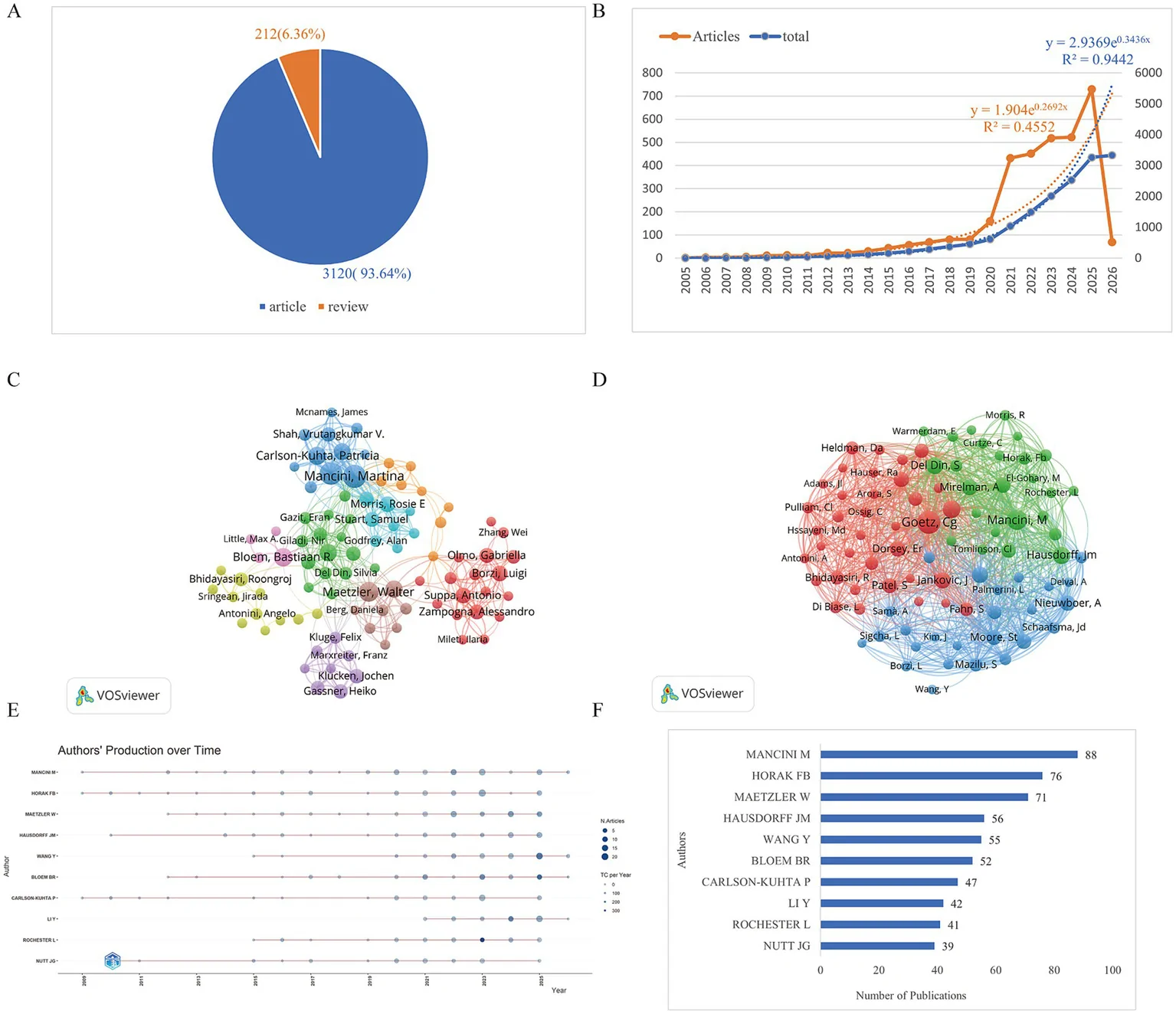
Publication trends and analysis of author networks and co-cited authors. **(A)** Literature type distribution; **(B)** Price’s Law of cumulative growth curve fitting analysis; **(C)** Co-author network and **(D)** Co-cited author clustering analysis. Each circle represents an author, with lines indicating collaborative ties. The different colors of the circles denote collaborative clusters among authors. Node sizes in this network reflect the frequency of co-citations. **(E)** Annual publication trends of top 10 authors. **(F)** Top 10 authors by publication output.

The co-author and co-cited author networks reveal a highly collaborative landscape centered on pioneers in objective motor assessment. M. Mancini (88 publications, 3,968 citations) and F. B. Horak (76 publications, 3,571 citations) emerged as the most prolific and highly cited authors (Table 1), with consistent publication outputs over time alongside other key contributors like W. Maetzler (Figures 2E,F). These researchers form dense collaborative clusters (Figures 2C,D) alongside other leading clinical researchers such as B. R. Bloem. This network structure indicates that foundational knowledge in this field is strongly anchored by experts in gait analysis and clinical movement disorders.

**Table 1:** Top 10 researchers in wearable device studies for Parkinson’s disease (2005–2026).

Rank	Author	h_index	g_index	m_index	TC	NP	PY_start	Articles	Articles fractionalized
1	MANCINI M	33	62	1.833	3,968	88	2009	88	14.1
2	HORAK FB	27	59	1.5	3,571	76	2009	76	11.2
3	BLOEM BR	26	52	1.733	3,225	52	2012	52	7.04
4	HAUSDORFF JM	25	48	1.471	2,333	56	2010	56	5.11
5	MAETZLER W	24	47	1.6	2,287	71	2012	71	7.02
6	ROCHESTER L	22	41	1.833	2,802	41	2015	41	3.6
7	CARLSON-KUHTA P	20	47	1.111	2,389	47	2009	47	6.38
8	NUTT JG	20	39	1.176	2,144	39	2010	39	5.62
9	WANG Y	20	43	1.667	1895	55	2015	55	8.14
10	CHIARI L	19	27	1.056	1,611	27	2009	27	2.86

### 3.2 Country/region and institutional contributions

As shown in Supplementary Table 2, The United States (U.S.) is the primary driver of global research in this domain, contributing the most publications (289 papers) and achieving the greatest overall academic impact (14,961 total citations). China ranked second in total output (206 publications), reflecting its rapid emergence in the digital health landscape. Longitudinal trends indicate that annual publications from these leading nations accelerated notably after 2019, with the U.S. exhibiting the most substantial growth (Figure 3A). Analysis of corresponding authorship and collaboration types (Figure 3B) reveals distinct regional strategies. The U.S. and China exhibit strong independent domestic research capabilities, characterized by a high proportion of single-country publications (SCP). In contrast, European nations—particularly Italy, Germany, and the United Kingdom—act as crucial hubs for international cooperation, demonstrating notably higher rates of multinational collaborative publications (MCP) within the global network (Figures 3C,D).

**Figure 3:**
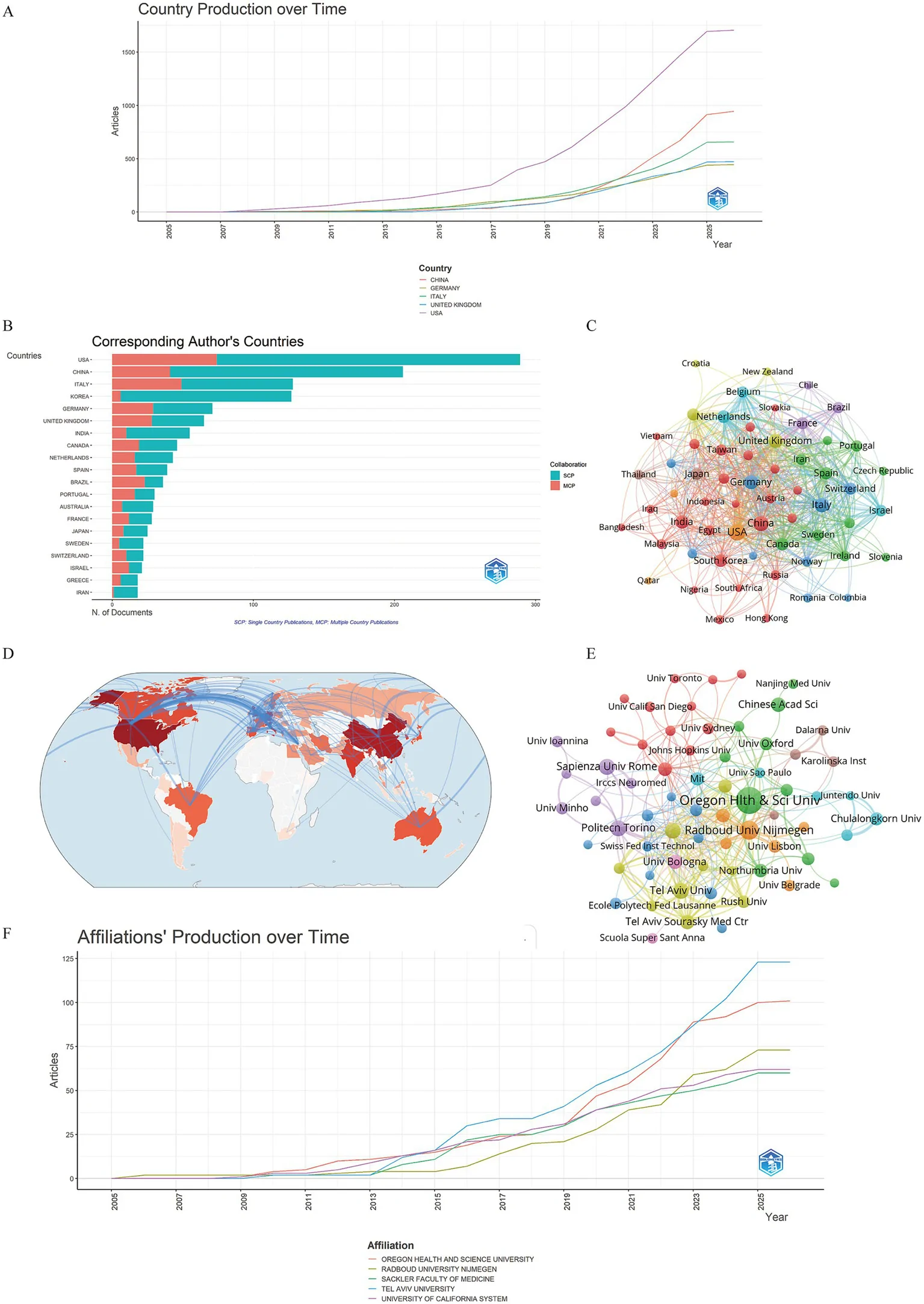
Visualization and analysis of Country/Region and institutional involvement. **(A)** Annual publication counts of the top 5 Country/Region. **(B)** the top 10 countries responsible for the number of studies (SCP, Single Country Publications; indicating that the authors of the article are from the same country; MCP, Multiple Country Publications, indicating that the author of the article is from multiple countries, that is, there is cooperation between countries). **(C)** Network map of national research output and cooperative relations. **(D)** Country/region Collaboration Map. Darker shades of red indicate higher collaboration rates, and the width of the link lines represents the strength of collaboration between two countries. **(E)** Institutional clustering analysis (each point represents an institution, the size of the node represents the number of publications, and the thickness of the lines between nodes represents the intensity of cooperation between institutions). **(F)** Annual publication counts of the Top 5 institutions.

At the institutional level, research output accelerated significantly after 2020 (Figure 3F). Tel Aviv University (123 articles) and Oregon Health and Science University (101 articles) emerged as the most prolific centers (Supplementary Table 3). The institutional collaboration network (Figure 3E) highlights these two universities as prominent central nodes, working closely with other high-output institutions including Radboud University Nijmegen, the University of California System, and Harvard University.

### 3.3 Multidisciplinary convergence across source journals

The source analysis reveals a strong cross-disciplinary convergence between biomedical engineering and clinical neurology. Technical and bioengineering journals, notably *Sensors* (311 publications, 9,075 citations), dominated the overall publication output and longitudinal growth trends (Figures 4A,B; Table 2). However, prominent clinical neurology journals, such as *Movement Disorders*, maintained highly influential citation impacts within the co-cited network (Figure 4D) despite contributing fewer than 50 primary research articles to this specific dataset (Table 2). Furthermore, the cited-journal network and dual-map overlay illustrate robust citation flows bridging technical clusters (e.g., sensors, physics, and bioengineering) with clinical domains (e.g., neurorehabilitation and movement disorders) (Figures 4C,E). This distribution highlights that while technological feasibility is rapidly advancing, rigorous clinical validation published in top-tier neurological journals remains comparatively scarce but highly impactful.

**Figure 4:**
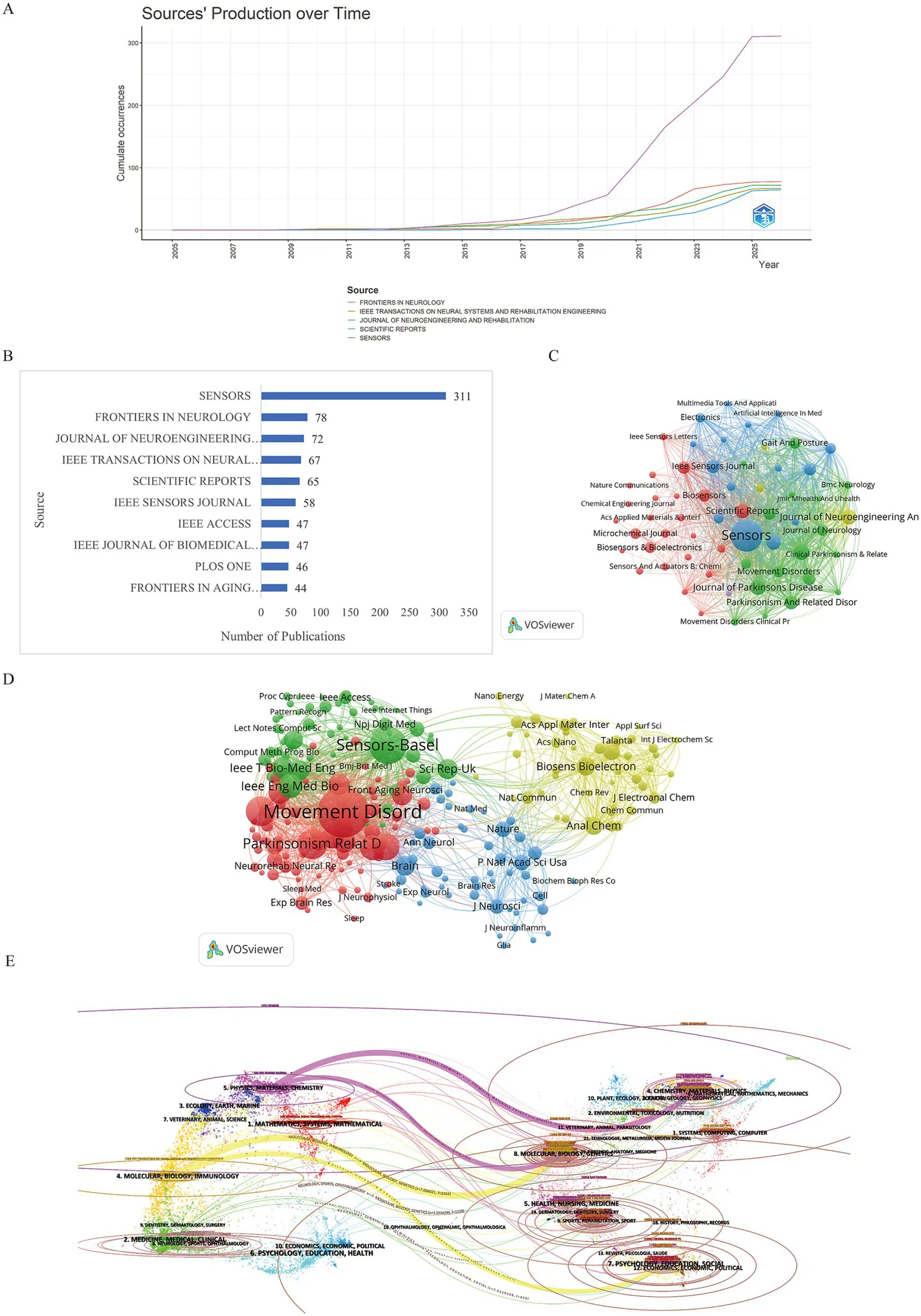
Visualization and analysis of journal involvement. **(A)** Annual publication counts of the top 5 journals. **(B)** Top 10 journals by publication volume. **(C)** Cited Journals network based on VOSviewer. **(D)** Co-Cited Journals Clustering Analysis based on VOSviewer. Each circle represents a Journal, with lines indicating collaborative ties. The different colors of the circles denote collaborative clusters among Journals. Node sizes in this network reflect the frequency of co-citations. **(E)** Journal Dual-Map Overlay Analysis: Dual-map visualization showing citing and cited journal relationships, with lines indicating citation flows and knowledge transfer.

**Table 2:** Top 10 journals by influence rank according to bibliometrix analysis (2005–2026).

Rank	Source	h_index	g_index	m_index	TC	NP	PY_start	Articles	JCR	IF
1	SENSORS	51	79	2.833	9,075	311	2009	311	Q2	3.5
2	JOURNAL OF NEUROENGINEERING AND REHABILITATION	31	57	2.067	3,311	72	2012	72	Q1	5.2
3	FRONTIERS IN NEUROLOGY	25	43	1.786	2,111	78	2013	78	Q2	2.8
4	IEEE JOURNAL OF BIOMEDICAL AND HEALTH INFORMATICS	24	47	2	2,372	47	2015	47	Q1	6.8
5	IEEE TRANSACTIONS ON NEURAL SYSTEMS AND REHABILITATION ENGINEERING	24	47	1.333	2,282	67	2009	67	Q1	5.2
6	MOVEMENT DISORDERS	24	43	1.143	2,870	43	2006	43	Q1	7.6
7	IEEE SENSORS JOURNAL	22	41	1.833	1768	58	2015	58	Q2	4.5
8	BIOSENSORS	21	37	3.5	4,097	37	2021	37	Q1	5.6
9	PLOS ONE	21	46	1.4	2,132	46	2012	46	Q2	2.6
10	SCIENTIFIC REPORTS	21	35	1.75	1,374	65	2015	65	Q1	3.9

### 3.4 Foundational knowledge and cited references

The most frequently cited publications establish the historical foundation of engineering validation in this field (Table 3). Foundational works by Patel et al. (2009) and Salarian et al. (2007b)—which hold the highest citation frequencies—focused on establishing the initial feasibility of quantifying core Parkinsonian features (Rovini et al., 2017) using novel ambulatory monitoring systems. Reference co-citation and CiteSpace clustering analyses further reflect this technological maturation (Figures 5A,D). Early structural clusters were predominantly anchored in fundamental “ambulatory monitoring” (#9), “inertial signal” acquisition (#4), and “gait detection” (#5). As demonstrated by the temporal distribution of these co-cited references (Figure 5B), the field systematically progressed from these early signal processing efforts toward more complex clinical applications, including home-based continuous assessments and intelligent diagnostic systems (e.g., cluster #0 “using machine learning”). This evolutionary trajectory is quantitatively supported by the references with the strongest citation bursts (Figure 5C), which highlight how early feasibility studies provided the methodological bedrock necessary for the recent shift toward AI-driven digital biomarkers and clinical decision support.

**Table 3:** Top 10 most cited publications based on bibliometrix analysis (2005–2026).

Rank	Author	Title	Journal	years	DOI	Total citations	Normalized TC
1	Shyamal Patel	Monitoring motor fluctuations in patients with Parkinson’s disease using wearable sensors	IEEE Trans Inf Technol Biomed	2009	10.1109/TITB.2009.2033471	101	7.72
2	Arash Salarian	Quantification of tremor and bradykinesia in Parkinson’s disease using a novel ambulatory monitoring system	IEEE Trans Biomed Eng	2007	10.1109/TBME.2006.886670	86	3.07
3	Steven T Moore	Ambulatory monitoring of freezing of gait in Parkinson’s disease	J Neurosci Methods	2008	10.1016/j.jneumeth.2007.08.023	79	4.25
4	Erika Rovini	How Wearable Sensors Can Support Parkinson’s Disease Diagnosis and Treatment: A Systematic Review	Front Neurosci	2017	10.3389/fnins.2017.00555	78	9.89
5	Walter Maetzler	Quantitative wearable sensors for objective assessment of Parkinson’s disease	Mov Disord	2013	10.1002/mds.25628	73	5.34
6	Marc Bächlin	Wearable assistant for Parkinson’s disease patients with the freezing of gait symptom	IEEE Trans Inf Technol Biomed	2010	10.1109/TITB.2009.2036165	68	3.96
7	Carolin Curtze	Levodopa Is a Double-Edged Sword for Balance and Gait in People With Parkinson’s Disease	Mov Disord	2015	10.1002/mds.26269	64	7.26
8	Johannes C M Schlachetzki	Wearable sensors objectively measure gait parameters in Parkinson’s disease	PLoS One	2017	10.1371/journal.pone.0183989	61	7.74
9	Silvia Del Din	Free-living monitoring of Parkinson’s disease: Lessons from the field	Mov Disord	2016	10.1002/mds.26718	52	5.08
10	Evanthia E Tripoliti	Automatic detection of freezing of gait events in patients with Parkinson’s disease	Comput Methods Programs Biomed	2013	10.1016/j.cmpb.2012.10.016	51	3.73

**Figure 5:**
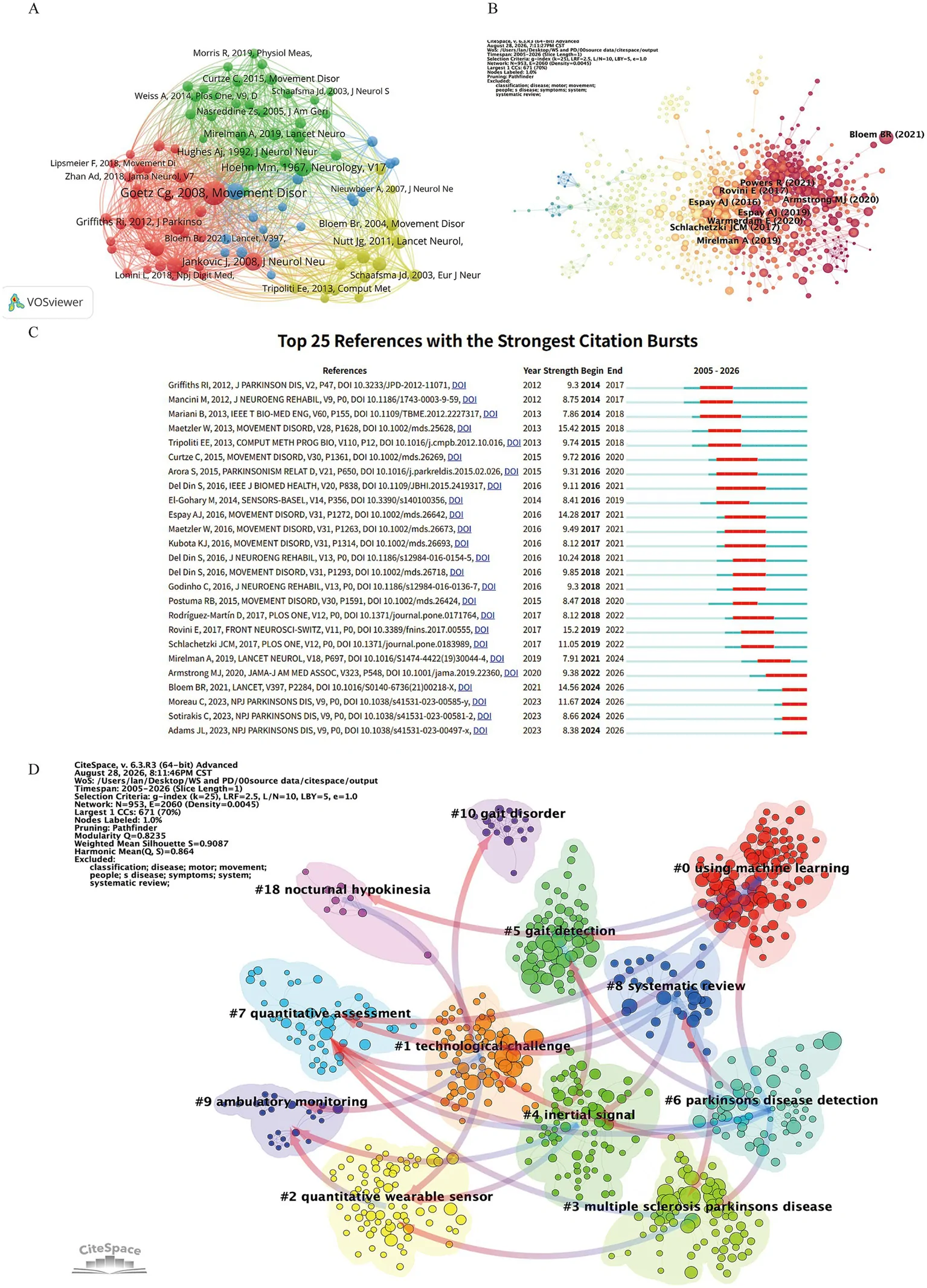
Visualization and analysis of references involvement. **(A)** Co-cited references clustering analysis based on VOSviewer. **(B)** Co-occurrence map of cited literature network based on CiteSpace. **(C)** Top 25 references with the strongest citation bursts. The blue lines indicate the time intervals, while red lines mark the periods of burst activity for each reference, showing the initial and final years of the citation burst duration. **(D)** Co-cited references clustering analysis based on CiteSpace.

### 3.5 Thematic clustering and evolutionary keyword bursts

Keyword co-occurrence networks (Figures 6A,D) revealed a structured thematic landscape spanning fundamental pathology, clinical assessment, and technological application. Cluster analysis further categorized these domains, demonstrating strong integration between clinical targets (e.g., “freezing of gait” [#4], “legged locomotion” [#12]) and digital monitoring innovations (e.g., “wearable sensors” [#2], “digital health technology” [#8]) (Figure 6C). Notably, a prominent cluster centered on “deep brain stimulation” (#1) emerged alongside traditional kinematic assessment clusters, underscoring the field’s expanding intersection with neuromodulation.

**Figure 6:**
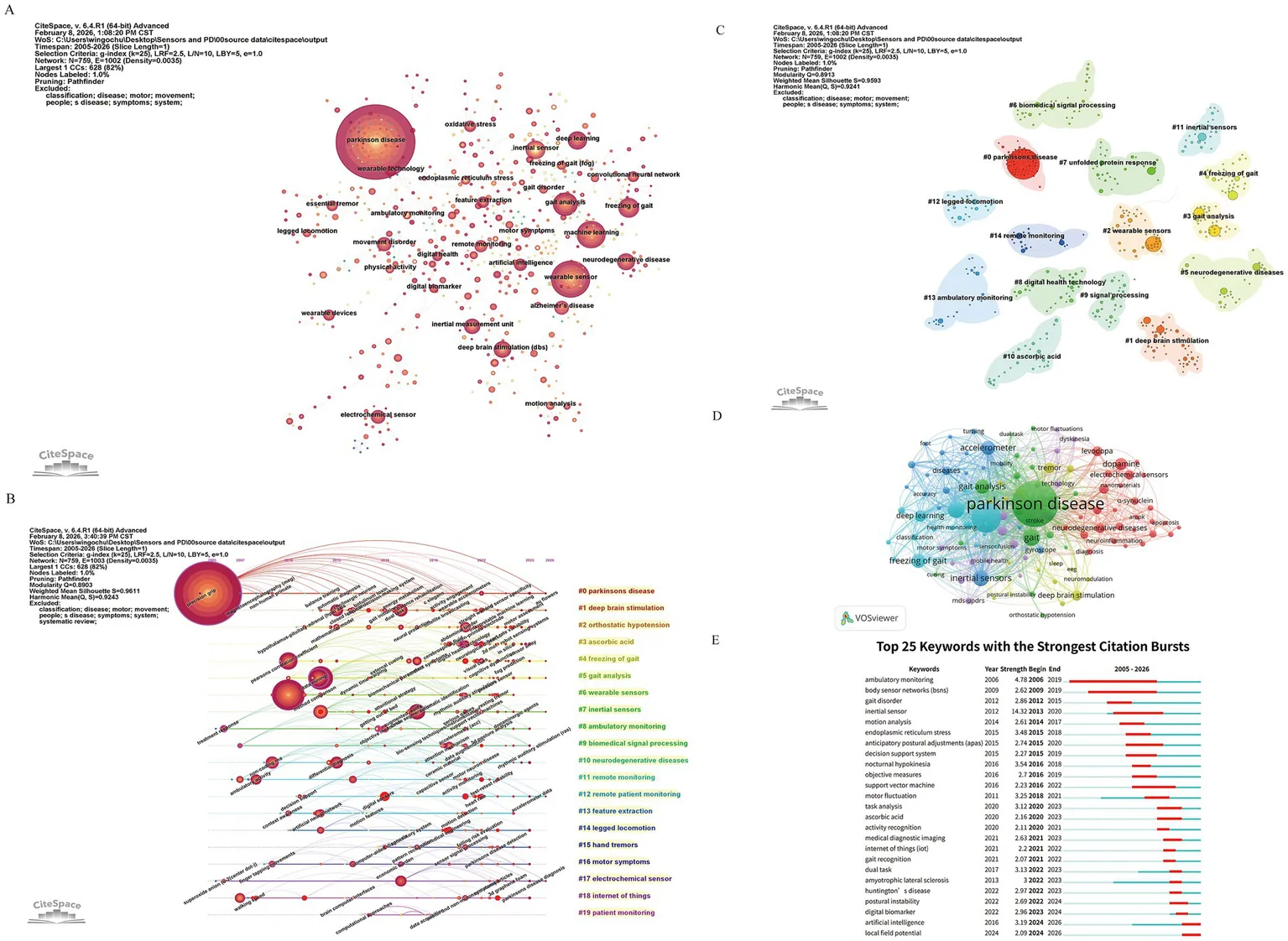
Visualization of keywords. **(A)** Author keywords map based on CiteSpace. **(B)** Timeline of keyword evolution based on CiteSpace. **(C)** Keyword clustering map based on CiteSpace. **(D)** Co-occurrence author keywords clustering analysis based on VOSviewer. **(E)** Top 25 references with the strongest citation bursts.

Keyword burst detection and timeline visualizations quantitatively mapped the chronological evolution of these research priorities (Figures 6B,E). The early developmental phase (2006–2014) was dominated by foundational hardware and feasibility concepts, marked by strong citation bursts for “ambulatory monitoring” and “inertial sensor” (burst strength 14.32). During the intermediate phase (2015–2020), the focus shifted toward algorithmic data processing and symptom classification, highlighted by emerging terms such as “support vector machine.” Crucially, the most recent period exhibits strong bursts for advanced analytics and physiological metrics, including “artificial intelligence,” “digital biomarker,” and “local field potential” (Figure 6E). This temporal trajectory, clearly visualized across the cluster timelines (Figure 6B), reflects the field’s ongoing transition from the passive, external tracking of motor fluctuations toward AI-driven data fusion and internal neurophysiological monitoring.

## 4 Discussion

### 4.1 Rapid expansion versus clinical readiness

The exponential growth in publication output, particularly post-2019, reflects a profound methodological convergence between biomedical engineering, AI, and clinical neurology. This momentum is driven by a critical clinical imperative: standard scheduled outpatient assessments fail to fully capture the complex, real-world symptom fluctuations inherent to PD. Wearable and sensor-based technologies promise to bridge this measurement gap by enabling continuous monitoring in free-living settings.

However, this bibliometric surge must be interpreted cautiously. High publication volume signifies research enthusiasm and technical feasibility, not necessarily proven clinical utility. As the literature expands, it reveals profound heterogeneity across hardware devices, signal-processing pipelines, and clinical validation designs. This lack of standardization highlights a major translational bottleneck: while the field is rapidly generating novel digital tools, it urgently requires rigorous, standardized clinical validation before these technologies can be routinely adopted into clinical decision-making.

### 4.2 Global collaboration as a catalyst for clinical translation

The geographic landscape of this field reflects a necessary convergence of engineering capacity and clinical infrastructure. While nations like the United States and China exhibit high independent research volumes, European countries form a remarkably dense multinational collaborative hub. This structure is driven by the inherently interdisciplinary demands of “digital phenotyping.” Developing robust sensor-based tools requires not only technical expertise in bioengineering but also deep integration with movement-disorder centers that can provide well-characterized patient cohorts. However, it is crucial to recognize that high bibliometric centrality primarily indicates publication visibility and robust citation networks, rather than widespread clinical implementation.

To bridge the gap between technically successful prototypes and clinically interpretable systems, future progress relies heavily on cross-regional data sharing and harmonized protocols (Del Din et al., 2016; Espay et al., 2019). Sensor-derived metrics are highly susceptible to clinical confounders, including disease stage, medication-induced fluctuations, comorbidities, and variations in device placement. Consequently, large-scale, multicenter collaborations are strictly necessary to mitigate single-center bias and validate these tools across diverse, real-world populations. Ultimately, global collaborative networks must shift their focus from merely expanding technological feasibility toward standardizing clinical validation frameworks.

### 4.3 Thematic evolution from engineering validation to digital monitoring

The co-citation, clustering, and keyword-burst results suggest a gradual thematic evolution from engineering validation and objective motor quantification toward AI-enabled digital biomarkers and remote monitoring. Earlier work focused on feasibility, inertial sensing, ambulatory monitoring, and objective measurement of gait, tremor, bradykinesia, dyskinesia, and FOG (Del Din et al., 2016; Maetzler et al., 2013; Rovini et al., 2017; Salarian et al., 2007a,b). This early emphasis was necessary because wearable and sensor-based monitoring technologies first had to establish whether clinically relevant motor phenomena could be captured reliably outside conventional examination settings. For instance, foundational studies successfully demonstrated that ambulatory gait monitors were sensitive enough to capture dynamic locomotor responses to levodopa therapy under real-world conditions (Moore et al., 2008). Furthermore, pioneer studies proved the capability of wearable sensor networks to automatically detect complex and episodic motor phenomena, such as the FOG, in real-time (Bächlin et al., 2010), while innovations like noncontact ultra-wideband systems enabled unobtrusive tremor assessment (Blumrosen et al., 2012). Research questions in this phase often centered on signal acquisition, sensor placement, algorithmic detection, and agreement with clinical scales or expert annotation with performance typically evaluated using measures such as sensitivity, specificity, accuracy, and agreement with clinician-derived outcomes.

More recent work places greater emphasis on machine learning, digital biomarkers, remote assessment, multimodal signals, and longitudinal monitoring (Bacanoiu et al., 2025; Mejia et al., 2025; Park et al., 2025). This shift suggests that the field is moving beyond isolated measurement tasks toward systems designed to characterize disease state, symptom variability, treatment response, and daily functioning over time. For example, recent advancements have increasingly utilized deep learning algorithms to translate continuous, free-living sensor data into clinically meaningful metrics, such as estimating MDS-UPDRS scores remotely (Adams et al., 2024; Liu et al., 2025). Importantly, the increasing diversity of digital assessment approaches has also generated a growing body of evidence regarding their diagnostic and monitoring performance (Cox and Mackett, 2024). Across wearable sensors, smartphone-based assessments, computer vision, and other digital modalities, studies have reported promising performance for detecting specific motor manifestations, including gait abnormalities, tremor, bradykinesia, dyskinesia, and freezing of gait (Ancona et al., 2022; Gregorčič and Georgiev, 2025; Rovini et al., 2017), as well as for estimating overall disease severity (Zhan et al., 2018). Reported performance measures include sensitivity, specificity, accuracy, area under the receiver operating characteristic curve (AUC), correlation with clinician-rated scales, and prediction errors for continuous severity estimation (di Biase et al., 2024; Silva de Lima et al., 2017; Zhan et al., 2018). However, direct comparison of these performance estimates remains challenging because studies differ in target symptoms, sensor configurations, datasets, reference standards, disease stages, and validation strategies (Godinho et al., 2016; Li et al., 2025; Silva de Lima et al., 2017). Consequently, high performance reported in individual studies should not be interpreted as evidence of generalizable clinical utility (Falkenburger et al., 2026; Virameteekul et al., 2026). Rather, the current literature suggests a transition from demonstrating technical feasibility toward establishing reproducible, clinically meaningful, and externally validated performance across diverse real-world populations and settings (Virameteekul et al., 2026; Youssef et al., 2026). Parallel to validating external sensors, our keyword clustering map and keyword burst analyses—particularly the prominent DBS cluster and the recent LFP burst (2024–2026)—highlight a critical shift toward adaptive neuromodulation (van Wijk et al., 2023). A primary focus is adaptive deep brain stimulation (aDBS), which fuses external kinematics with internal LFP signals (Melbourne et al., 2023) to dynamically tailor stimulation to symptom fluctuations (Dixon et al., 2026). However, translating these closed-loop architectures into clinical practice entails overcoming technical hurdles, such as real-time artifact rejection and multimodal data integration (Hammer et al., 2022). Consequently, establishing their safety and long-term efficacy demands rigorous clinical validation (Marceglia et al., 2022).

Furthermore, by capturing high-frequency motor fluctuations—such as transient ON/OFF states and medication-induced dyskinesia—that are notoriously difficult to assess during brief clinical encounters, these continuous monitoring paradigms enable data-driven therapeutic adjustments. Ultimately, this comprehensive digital phenotyping lays the essential groundwork for precision medicine and personalized care strategies in PD (Klucken et al., 2018; Powers et al., 2021). Concurrently, the development of integrated web-based platforms has begun to bridge the gap between continuous remote data collection and clinician interfaces, paving the way for real-time personalized management (Cubo and Delgado-López, 2022; Guan et al., 2021). However, in this context, digital biomarkers should be interpreted cautiously: bibliometric prominence indicates that the concept has become a research focus, but it does not by itself establish analytical validity, clinical validity, or clinical utility (Goldsack et al., 2020). For these measures to become useful in practice, algorithms must be evaluated across devices, settings, patient subgroups, and real-world contexts, with transparent reporting of performance, uncertainty, and failure modes.

The keyword co-occurrence and clustering results indicate that the field is broadening rather than following a single linear pathway. The convergence of terms such as “gait analysis,” “wearable sensors,” “inertial sensors,” “deep brain stimulation”, “digital health technology”, “ambulatory monitoring”, and “remote monitoring” reflects overlapping research communities rather than isolated thematic silos. This confluence of motor assessment, signal processing, digital health, electrophysiological monitoring, and biomarker-oriented terminology suggests that wearable and sensor-based technologies are increasingly embedded within a comprehensive ecosystem of PD assessment (Bacanoiu et al., 2025; Brzenczek et al., 2024; Zhang et al., 2024). While these bibliometric patterns indicate a profound shift in research priorities, they should not be interpreted as evidence that these digital tools are already standardized or seamlessly integrated into routine clinical care (Bougea, 2024; Espay et al., 2016). Bridging this gap between research feasibility and clinical implementation requires concerted efforts in regulatory science (Sacks and Kunkoski, 2021). Ongoing initiatives by international task forces—such as the qualification of digital endpoints by regulatory bodies and collaborative frameworks like the Critical Path for Parkinson’s Consortium—remain essential to establish the unified analytical and clinical validity required for widespread clinical adoption (Espay et al., 2019; Hill et al., 2025; Stephenson et al., 2020; Stephenson et al., 2021).

### 4.4 Future directions and translational requirements

The next stage of research will likely depend less on additional isolated sensor studies than on establishing reproducible, clinically interpretable, and workflow-compatible systems. In particular, the heterogeneity in reported performance identified in the current literature highlights the need for standardized performance benchmarks and validation frameworks. Key priorities include standardizing data collection and reporting protocols, validating algorithms in real-world and multicenter settings, integrating outputs into clinical decision-making workflows, and combining movement, physiologic, neurophysiologic, imaging, and patient-reported data when appropriate. Future studies should therefore move beyond reporting isolated accuracy, sensitivity, specificity, or AUC values and adopt more comprehensive evaluation frameworks that also consider reliability, calibration, responsiveness to longitudinal change, uncertainty, and clinically meaningful thresholds. Standardization is especially important for device placement, sampling frequency, preprocessing steps, task definitions, annotation procedures, missing-data handling, and outcome reporting. Without this methodological alignment, results across studies may remain difficult to compare, even when they address similar symptoms or use similar sensing modalities.

Real-world validation is another central requirement. Many sensor-based studies demonstrate feasibility or classification performance under controlled or semi-controlled conditions, but clinical translation requires evidence that measurements remain reliable during daily activities, across medication cycles, and in diverse patient populations (Evers et al., 2020; Oyama et al., 2023). Accordingly, the promising performance reported in individual studies should be followed by independent external validation to determine whether such performance is reproducible across devices, healthcare settings, disease stages, and patient subgroups. Validation should therefore include multicenter cohorts, longitudinal follow-up, clinically meaningful endpoints, and comparison with established clinical assessments where appropriate. Importantly, longitudinal validation should assess not only cross-sectional agreement with clinical scales but also sensitivity to clinically meaningful changes in disease severity, treatment response, and symptom fluctuations. Studies should also define how digital outputs would affect clinical decisions. A metric that is statistically accurate but difficult to interpret, burdensome to review, or disconnected from treatment decisions is unlikely to be adopted in routine care.

Workflow integration will determine whether wearable and sensor-based monitoring technologies become usable clinical tools rather than parallel research outputs. Thus, demonstrating high analytical or diagnostic performance should be regarded as an intermediate step rather than the endpoint of translational development. Clinicians need concise, interpretable summaries rather than unfiltered streams of sensor data, and patients need systems that minimize burden, protect privacy, and provide clear value. Future platforms should therefore consider alert thresholds, longitudinal dashboards, uncertainty estimates, integration with clinical documentation, and mechanisms for responding to clinically relevant changes. Prospective studies should increasingly evaluate whether incorporating digital measures into clinical workflows improves treatment decisions, monitoring efficiency, or patient outcomes, thereby providing direct evidence of clinical utility. These requirements are methodological and operational as much as technological.

Multimodal data fusion and closed-loop applications represent important but higher-risk frontiers. Combining movement signals with physiologic, neurophysiologic, imaging, and patient-reported data may improve characterization of PD heterogeneity and treatment response, but it also increases the complexity of data governance, model interpretation, and validation. The integration of wearable and sensor-based monitoring technologies with EEG, local field potentials, and adaptive neuromodulation may support future closed-loop approaches (Habets et al., 2018), but this direction requires cautious validation of safety, interpretability, generalizability, and clinical utility before strong translational claims can be made (Bronte-Stewart et al., 2020; di Biase et al., 2021; Guidetti et al., 2025). For bibliometric interpretation, these emerging themes should therefore be viewed not simply as evidence of technological progress, but as indicators of a broader shift toward standardized, externally validated, longitudinally informative, and clinically actionable digital assessment. These topics mark emerging research frontiers, not established standards of care.

### 4.5 Limitations

Several limitations should be considered. First, restricting the analysis to English-language publications may have introduced language bias and resulted in the exclusion of relevant studies published in other languages. Second, although WoSCC and Scopus are widely used bibliographic databases, reliance on these two sources may have resulted in incomplete coverage of the literature, as different bibliographic databases vary in their coverage and disciplinary focus. Relevant studies indexed in other databases, particularly PubMed, Embase, and IEEE Xplore, may therefore not have been fully captured (AlRyalat et al., 2019; Bramer et al., 2016; Gusenbauer, 2024). Third, the search strategy was based on broad concepts related to wearable technologies, sensors, and monitoring; thus, studies using highly specific device names or terminology not captured by the predefined search terms may have been missed. Fourth, citation-based indicators may be influenced by topic popularity, author visibility, publication age, and citation practices and therefore do not necessarily reflect methodological quality or clinical impact. Accordingly, bibliometric findings should be interpreted as measures of research activity and influence rather than direct evidence of clinical effectiveness. Fifth, because the search was conducted on February 2, 2026, the 2026 publication record was necessarily incomplete. Finally, despite extensive data curation, duplicate removal, and synonym merging, potential bias arising from the clustering algorithms and parameter settings used in the bibliometric software cannot be completely excluded.

## 5 Conclusion

This study provides science-mapping and bibliometric evidence on the global development of wearable and sensor-based monitoring technologies in Parkinson’s disease. The findings indicate rapid growth in research activity, an evolving collaboration landscape, and a thematic shift toward AI-enabled digital biomarkers, remote and multimodal monitoring, and cautious exploration of closed-loop applications. Future research should emphasize standardization, multicenter real-world validation, clinical workflow integration, multimodal data fusion, and careful evaluation of translational readiness before these technologies are interpreted as established clinical tools.

## Glossary

AI: artificial intelligence
APAs: anticipatory postural adjustments
AUC: area under the receiver operating characteristic curve
BSN: body sensor networks
EEG: electroencephalography
FOG: freezing of gait
IF: impact factor
JCR: Journal Citation Reports
LFPs: local field potentials
LLR: log-likelihood ratio
MCP: multiple-country publications
MDS-UPDRS: Movement Disorder Society-sponsored revision of the Unified Parkinson’s Disease Rating Scale
NC: number of citations
NP: number of publications
PD: Parkinson’s disease
SCP: single-country publications
TC: total citations
U.K.: United Kingdom
U.S.: United States
WoSCC: Web of Science Core Collection

## References

[ref1] Adams J. L. Kangarloo T. Gong Y. Khachadourian V. Tracey B. Volfson D. . (2024). Using a smartwatch and smartphone to assess early Parkinson's disease in the WATCH-PD study over 12 months. NPJ Parkinsons Dis. 10:112. doi: 10.1038/s41531-024-00721-2, 38866793 PMC11169239

[ref2] AlRyalat S. A. S. Malkawi L. W. Momani S. M. (2019). Comparing bibliometric analysis using PubMed, Scopus, and web of science databases. J. Vis. Exp. 58494. doi: 10.3791/58494 31710021

[ref3] Ancona S. Faraci F. D. Khatab E. Fiorillo L. Gnarra O. Nef T. . (2022). Wearables in the home-based assessment of abnormal movements in Parkinson's disease: a systematic review of the literature. J. Neurol. 269, 100–110. doi: 10.1007/s00415-020-10350-3, 33409603

[ref4] Aria M. Cuccurullo C. (2017). Bibliometrix: an R-tool for comprehensive science mapping analysis. J. Informetr. 11, 959–975. doi: 10.1016/j.joi.2017.08.007

[ref5] Bacanoiu M. V. Rusu L. Marin M. I. Piele D. Rusu M. R. Danoiu R. . (2025). Digital health in Parkinson's disease and atypical parkinsonism-new frontiers in motor function and physical activity assessment: review. J. Clin. Med. 14:4140. doi: 10.3390/jcm14124140, 40565895 PMC12194145

[ref6] Bächlin M. Plotnik M. Roggen D. Maidan I. Hausdorff J. M. Giladi N. . (2010). Wearable assistant for Parkinson's disease patients with the freezing of gait symptom. IEEE Trans. Inf. Technol. Biomed. 14, 436–446. doi: 10.1109/titb.2009.2036165, 19906597

[ref7] Blumrosen G. Uziel M. Rubinsky B. Porrat D. (2012). Noncontact tremor characterization using low-power wideband radar technology. I.E.E.E. Trans. Biomed. Eng. 59, 674–686. doi: 10.1109/tbme.2011.2177977, 22155937

[ref8] Bougea A. (2024). Digital biomarkers in Parkinson's disease. Adv. Clin. Chem. 123, 221–253. doi: 10.1016/bs.acc.2024.06.005, 39181623

[ref9] Bougea A. (2025). Application of wearable sensors in Parkinson’s disease: state of the art. J. Sens. Actuator Netw. 14:23. doi: 10.3390/jsan14020023

[ref10] Bramer W. M. Giustini D. Kramer B. M. (2016). Comparing the coverage, recall, and precision of searches for 120 systematic reviews in Embase, MEDLINE, and Google scholar: a prospective study. Syst. Rev. 5:39. doi: 10.1186/s13643-016-0215-7, 26932789 PMC4772334

[ref11] Bronte-Stewart H. M. Petrucci M. N. O'Day J. J. Afzal M. F. Parker J. E. Kehnemouyi Y. M. . (2020). Perspective: evolution of control variables and policies for closed-loop deep brain stimulation for Parkinson's disease using bidirectional deep-brain-computer interfaces. Front. Hum. Neurosci. 14:353. doi: 10.3389/fnhum.2020.00353 PMC7489234 33061899

[ref12] Brzenczek C. Klopfenstein Q. Hähnel T. Sapienza S. Klucken J. Fröhlich H. . (2024). Integrating digital gait data with metabolomics and clinical data to predict outcomes in Parkinson's disease. NPJ Digit. Med. 7:235. doi: 10.1038/s41746-024-01236-z, 39242660 PMC11379877

[ref13] Burq M. Rainaldi E. Ho K. C. Chen C. Bloem B. R. Evers L. J. W. . (2022). Virtual exam for Parkinson's disease enables frequent and reliable remote measurements of motor function. NPJ Digit. Med. 5:65. doi: 10.1038/s41746-022-00607-8, 35606508 PMC9126938

[ref14] Chen C. Dubin R. Kim M. C. (2014). Emerging trends and new developments in regenerative medicine: a scientometric update (2000 - 2014). Expert. Opin. Biol. Ther. 14, 1295–1317. doi: 10.1517/14712598.2014.920813, 25077605

[ref15] Cox C. L. Mackett A. J. (2024). Wearable technologies for Parkinson's disease: exploring their clinical potential. Touchreviews Neurol. 20, 13–19. doi: 10.17925/usn.2024.20.2.4

[ref16] Cubo E. Delgado-López P. D. (2022). Telemedicine in the Management of Parkinson's disease: achievements, challenges, and future perspectives. Brain Sci. 12:1735. doi: 10.3390/brainsci12121735, 36552194 PMC9775481

[ref17] Del Din S. Godfrey A. Mazzà C. Lord S. Rochester L. (2016). Free-living monitoring of Parkinson's disease: lessons from the field. Mov. Disord. 31, 1293–1313. doi: 10.1002/mds.26718, 27452964

[ref18] di Biase L. Pecoraro P. M. Pecoraro G. Shah S. A. Di Lazzaro V. (2024). Machine learning and wearable sensors for automated Parkinson's disease diagnosis aid: a systematic review. J. Neurol. 271, 6452–6470. doi: 10.1007/s00415-024-12611-x, 39143345

[ref19] di Biase L. Tinkhauser G. Martin Moraud E. Caminiti M. L. Pecoraro P. M. Di Lazzaro V. (2021). Adaptive, personalized closed-loop therapy for Parkinson's disease: biochemical, neurophysiological, and wearable sensing systems. Expert. Rev. Neurother. 21, 1371–1388. doi: 10.1080/14737175.2021.2000392, 34736368

[ref20] Dixon T. C. Strandquist G. Zeng A. Frączek T. Bechtold R. Lawrence D. . (2026). Movement-responsive deep brain stimulation for Parkinson's disease using a remotely optimized neural decoder. Nat. Biomed. Eng. 10, 110–124. doi: 10.1038/s41551-025-01438-0, 40579487

[ref21] Espay A. J. Bonato P. Nahab F. B. Maetzler W. Dean J. M. Klucken J. . (2016). Technology in Parkinson's disease: challenges and opportunities. Mov. Disord. 31, 1272–1282. doi: 10.1002/mds.26642 PMC5014594 27125836

[ref22] Espay A. J. Hausdorff J. M. Sánchez-Ferro Á. Klucken J. Merola A. Bonato P. . (2019). A roadmap for implementation of patient-centered digital outcome measures in Parkinson's disease obtained using mobile health technologies. Mov. Disord. 34, 657–663. doi: 10.1002/mds.27671, 30901495 PMC6520192

[ref23] Evers L. J. Raykov Y. P. Krijthe J. H. Silva de Lima A. L. Badawy R. Claes K. . (2020). Real-life gait performance as a digital biomarker for motor fluctuations: the Parkinson@home validation study. J. Med. Internet Res. 22:e19068. doi: 10.2196/19068, 33034562 PMC7584982

[ref24] Falkenburger B. Fanciulli A. Pötter-Nerger M. van Eimeren T. Classen J. Höglinger G. . (2026). Clinical utility of digital technologies in Parkinson's disease. J. Neural Transm. (Vienna) 133, 297–307. doi: 10.1007/s00702-025-03062-3, 41222710 PMC12855330

[ref25] Godinho C. Domingos J. Cunha G. Santos A. T. Fernandes R. M. Abreu D. . (2016). A systematic review of the characteristics and validity of monitoring technologies to assess Parkinson's disease. J. Neuroeng. Rehabil. 13:24. doi: 10.1186/s12984-016-0136-7, 26969628 PMC4788909

[ref26] Goetz C. G. Tilley B. C. Shaftman S. R. Stebbins G. T. Fahn S. Martinez-Martin P. . (2008). Movement Disorder Society-sponsored revision of the unified Parkinson's disease rating scale (MDS-UPDRS): scale presentation and clinimetric testing results. Mov. Disord. 23, 2129–2170. doi: 10.1002/mds.22340 19025984

[ref27] Goldsack J. C. Coravos A. Bakker J. P. Bent B. Dowling A. V. Fitzer-Attas C. . (2020). Verification, analytical validation, and clinical validation (V3): the foundation of determining fit-for-purpose for biometric monitoring technologies (BioMeTs). NPJ Digit. Med. 3:55. doi: 10.1038/s41746-020-0260-4, 32337371 PMC7156507

[ref28] Gorce P. Jacquier-Bret J. (2026). Current trends in AI gait analysis for the detection and assessment of Parkinson's disease severity: systematic review and Meta-analysis of performance using logit transformation. Healthcare (Basel) 14:1820. doi: 10.3390/healthcare14131820, 42450835 PMC13362068

[ref29] Gregorčič M. Georgiev D. (2025). The usefulness of wearable sensors for detecting freezing of gait in Parkinson's disease: a systematic review. Sensors (Basel) 25:5101. doi: 10.3390/s25165101, 40871963 PMC12389820

[ref30] Guan I. Trabilsy M. Barkan S. Malhotra A. Hou Y. Wang F. . (2021). Comparison of the Parkinson's KinetiGraph to off/on levodopa response testing: single center experience. Clin. Neurol. Neurosurg. 209:106890. doi: 10.1016/j.clineuro.2021.106890, 34455169

[ref31] Guidetti M. Bocci T. De Pedro Del Álamo M. Deuschl G. Fasano A. Martinez-Fernandez R. . (2025). Will adaptive deep brain stimulation for Parkinson's disease become a real option soon? A Delphi consensus study. NPJ Parkinson. Dis. 11:110. doi: 10.1038/s41531-025-00974-5 PMC12052990 40325017

[ref32] Gusenbauer M. (2024). Beyond Google scholar, Scopus, and web of science: an evaluation of the backward and forward citation coverage of 59 databases' citation indices. Res. Synth. Methods 15, 802–817. doi: 10.1002/jrsm.1729, 38877607

[ref33] Habets J. G. V. Heijmans M. Kuijf M. L. Janssen M. L. F. Temel Y. Kubben P. L. (2018). An update on adaptive deep brain stimulation in Parkinson's disease. Mov. Disord. 33, 1834–1843. doi: 10.1002/mds.115, 30357911 PMC6587997

[ref34] Hammer L. H. Kochanski R. B. Starr P. A. Little S. (2022). Artifact characterization and a multipurpose template-based offline removal solution for a sensing-enabled deep brain stimulation device. Stereotact. Funct. Neurosurg. 100, 168–183. doi: 10.1159/000521431, 35130555 PMC9064887

[ref35] Hill D. L. Carroll C. Belfiore-Oshan R. Stephenson D. (2025). Aligning with regulatory agencies for the use of digital health technologies in drug development: a case study from Parkinson's disease. Front. Digit. Health 7:1415202. doi: 10.3389/fdgth.2025.1415202, 41208852 PMC12589013

[ref36] Huang T. Li M. Huang J. (2023). Recent trends in wearable device used to detect freezing of gait and falls in people with Parkinson's disease: a systematic review. Front. Aging Neurosci. 15:1119956. doi: 10.3389/fnagi.2023.1119956, 36875701 PMC9975590

[ref37] Janssen Daalen J. M. van den Bergh R. Prins E. M. Moghadam M. S. C. van den Heuvel R. Veen J. . (2024). Digital biomarkers for non-motor symptoms in Parkinson's disease: the state of the art. NPJ Digit. Med. 7:186. doi: 10.1038/s41746-024-01144-2, 38992186 PMC11239921

[ref38] Klucken J. Krüger R. Schmidt P. Bloem B. R. (2018). Management of Parkinson’s disease 20 years from now: towards digital health pathways. J. Parkinsons Dis. 8, S85–S94. doi: 10.3233/JPD-181519, 30584171 PMC6311358

[ref39] Li H. Zecca M. Huang J. (2025). Evaluating the utility of wearable sensors for the early diagnosis of Parkinson disease: systematic review. J. Med. Internet Res. 27:e69422. doi: 10.2196/69422, 40690754 PMC12322615

[ref40] Liu X. Zhang X. Li J. Pan W. Sun Y. Lin S. . (2025). Automated UPDRS gait scoring using wearable sensor fusion and deep learning. Bioengineering (Basel) 12. doi: 10.3390/bioengineering12070686, 40722378 PMC12292898

[ref41] Maetzler W. Domingos J. Srulijes K. Ferreira J. J. Bloem B. R. (2013). Quantitative wearable sensors for objective assessment of Parkinson's disease. Mov. Disord. 28, 1628–1637. doi: 10.1002/mds.25628, 24030855

[ref42] Majumder S. Mondal T. Deen M. J. (2017). Wearable sensors for remote health monitoring. Sensors (Basel) 17. doi: 10.3390/s17010130, 28085085 PMC5298703

[ref43] Marceglia S. Conti C. Svanidze O. Foffani G. Lozano A. M. Moro E. . (2022). Double-blind cross-over pilot trial protocol to evaluate the safety and preliminary efficacy of long-term adaptive deep brain stimulation in patients with Parkinson's disease. BMJ Open 12:e049955. doi: 10.1136/bmjopen-2021-049955, 34980610 PMC8724732

[ref44] Mejia A. C. Sapienza S. Paccoud I. Alcock L. Brown P. Gaßner H. . (2025). Consensus on the clinical utility of digital mobility outcomes for personalized clinical decision support in Parkinson's disease. Neurol. Res. Pract. 7:66. doi: 10.1186/s42466-025-00426-8, 40968373 PMC12447593

[ref45] Melbourne J. A. Kehnemouyi Y. M. O'Day J. J. Wilkins K. B. Gala A. S. Petrucci M. N. . (2023). Kinematic adaptive deep brain stimulation for gait impairment and freezing of gait in Parkinson's disease. Brain Stimul. 16, 1099–1101. doi: 10.1016/j.brs.2023.07.003, 37429355 PMC12673813

[ref46] Moore S. T. MacDougall H. G. Gracies J. M. Ondo W. G. (2008). Locomotor response to levodopa in fluctuating Parkinson's disease. Exp. Brain Res. 184, 469–478. doi: 10.1007/s00221-007-1113-y, 17828529

[ref47] Oyama G. Burq M. Hatano T. Marks W. J. Jr. Kapur R. Fernandez J. . (2023). Analytical and clinical validity of wearable, multi-sensor technology for assessment of motor function in patients with Parkinson's disease in Japan. Sci. Rep. 13:3600. doi: 10.1038/s41598-023-29382-6 PMC10015076 36918552

[ref48] Park H. Youm C. Cheon S. M. Kim B. Choi H. Hwang J. . (2025). Using machine learning to identify Parkinson's disease severity subtypes with multimodal data. J. Neuroeng. Rehabil. 22:126. doi: 10.1186/s12984-025-01648-2, 40457336 PMC12131740

[ref49] Patel S. Lorincz K. Hughes R. Huggins N. Growdon J. Standaert D. . (2009). Monitoring motor fluctuations in patients with Parkinson's disease using wearable sensors. IEEE Trans. Inf. Technol. Biomed. 13, 864–873. doi: 10.1109/titb.2009.2033471, 19846382 PMC5432434

[ref50] Powers R. Etezadi-Amoli M. Arnold E. M. Kianian S. Mance I. Gibiansky M. . (2021). Smartwatch inertial sensors continuously monitor real-world motor fluctuations in Parkinson's disease. Sci. Transl. Med. 13. doi: 10.1126/scitranslmed.abd7865, 33536284

[ref51] Qi W. Shen S. Dong C. Zhao M. Zang S. Zhu X. . (2025). Digital biomarkers for Parkinson disease: bibliometric analysis and a scoping review of deep learning for freezing of gait. J. Med. Internet Res. 27:e71560. doi: 10.2196/71560, 40392578 PMC12134701

[ref52] Rovini E. Maremmani C. Cavallo F. (2017). How wearable sensors can support Parkinson's disease diagnosis and treatment: a systematic review. Front. Neurosci. 11:555. doi: 10.3389/fnins.2017.00555, 29056899 PMC5635326

[ref53] Rovini E. Maremmani C. Cavallo F. (2019). Automated systems based on wearable sensors for the Management of Parkinson's disease at home: a systematic review. Telemed. J. E Health 25, 167–183. doi: 10.1089/tmj.2018.0035, 29969384

[ref54] Sacks L. Kunkoski E. (2021). Digital health technology to measure drug efficacy in clinical trials for Parkinson's disease: a regulatory perspective. J. Parkinsons Dis. 11, S111–s115. doi: 10.3233/jpd-202416, 33459666 PMC8385502

[ref55] Salarian A. Russmann H. Vingerhoets F. J. Burkhard P. R. Aminian K. (2007a). Ambulatory monitoring of physical activities in patients with Parkinson's disease. I.E.E.E. Trans. Biomed. Eng. 54, 2296–2299. doi: 10.1109/tbme.2007.896591, 18075046

[ref56] Salarian A. Russmann H. Wider C. Burkhard P. R. Vingerhoets F. J. Aminian K. (2007b). Quantification of tremor and bradykinesia in Parkinson's disease using a novel ambulatory monitoring system. I.E.E.E. Trans. Biomed. Eng. 54, 313–322. doi: 10.1109/tbme.2006.886670, 17278588

[ref57] Silva de Lima A. L. Evers L. J. W. Hahn T. Bataille L. Hamilton J. L. Little M. A. . (2017). Freezing of gait and fall detection in Parkinson's disease using wearable sensors: a systematic review. J. Neurol. 264, 1642–1654. doi: 10.1007/s00415-017-8424-0, 28251357 PMC5533840

[ref58] Stephenson D. Alexander R. Aggarwal V. Badawy R. Bain L. Bhatnagar R. . (2020). Precompetitive consensus building to facilitate the use of digital health technologies to support Parkinson disease drug development through regulatory science. Digit. Biomark. 4, 28–49. doi: 10.1159/000512500 PMC7768153 33442579

[ref59] Stephenson D. Badawy R. Mathur S. Tome M. Rochester L. (2021). Digital progression biomarkers as novel endpoints in clinical trials: a multistakeholder perspective. J. Parkinsons Dis. 11, S103–s109. doi: 10.3233/jpd-202428, 33579873 PMC8385507

[ref60] Synnestvedt M. B. Chen C. Holmes J. H. (2005). CiteSpace II: visualization and knowledge discovery in bibliographic databases. AMIA Annu. Symp. Proc. 2005, 724–728. PMC1560567 16779135

[ref61] van Eck N. J. Waltman L. (2010). Software survey: VOSviewer, a computer program for bibliometric mapping. Scientometrics 84, 523–538. doi: 10.1007/s11192-009-0146-3, 20585380 PMC2883932

[ref62] van Wijk B. C. M. de Bie R. M. A. Beudel M. (2023). A systematic review of local field potential physiomarkers in Parkinson's disease: from clinical correlations to adaptive deep brain stimulation algorithms. J. Neurol. 270, 1162–1177. doi: 10.1007/s00415-022-11388-1, 36209243 PMC9886603

[ref63] Virameteekul S. Shin C. Hirczy S. S. Sarva H. Paul S. S. Maetzler W. . (2026). Assessing digital health technologies for outcome measurement in Parkinson's disease drug trials: a systematic review. Mov. Disord. 41, 40–62. doi: 10.1002/mds.70097 41175008

[ref64] Youssef H. Sabıroğlu M. Mahmoud Abdel-Rahman S. Hasanin T. Manay M. Gönül M. N. . (2026). Can wearable sensor based measures of gait accurately reflect Parkinson's disease severity? A systematic review and meta-analysis. Parkinsonism Relat. Disord. 142:108108. doi: 10.1016/j.parkreldis.2025.108108, 41233205

[ref65] Zhan A. Mohan S. Tarolli C. Schneider R. B. Adams J. L. Sharma S. . (2018). Using smartphones and machine learning to quantify Parkinson disease severity: the Mobile Parkinson disease score. JAMA Neurol. 75, 876–880. doi: 10.1001/jamaneurol.2018.0809, 29582075 PMC5885192

[ref66] Zhang W. Ling Y. Chen Z. Ren K. Chen S. Huang P. . (2024). Wearable sensor-based quantitative gait analysis in Parkinson's disease patients with different motor subtypes. NPJ Digit. Med. 7:169. doi: 10.1038/s41746-024-01163-z, 38926552 PMC11208588

