# Supplementary material for: Digital health meets Parkinson’s disease: a bibliometric exploration of wearable sensors research and emerging frontiers (2005–2026)
Source: Front Neuroinform. 2026 Oct 8;20:1937585. doi: 10.3389/fninf.2026.1937585 (PMC13687709; doi:10.3389/fninf.2026.1937585)
Supplement: Supplementary file 1 [file Data_Sheet_1.docx]

**Supplemental Tables**

**Supplementary Table 1.** Bibliometric Overview of Research Publications on Wearable Devices for Parkinson's Disease

**Supplementary Table 2.** Most Relevant Countries by Corresponding Author Contributions (2005–2026)

**Supplementary Table 3**. Top 10 Affiliations and Articles in Wearable Device Research Publications for Parkinson's Disease (2005–2026)

**Supplementary Table 4**. Original search strategy for wearable and sensor-based monitoring technologies in Parkinson's disease

**Supplementary Table 1** Bibliometric Overview of Research Publications on Wearable Devices for Parkinson's Disease (2005–2026)

| **Description** | **Results** |
| --- | --- |
| MAIN INFORMATION ABOUT DATA |  |
| Timespan | 2005:2026 |
| Sources (Journals, Books, etc) | 803 |
| Documents | 3332 |
| Annual Growth Rate % | 22.34 |
| Document Average Age | 3.86 |
| Average citations per doc | 36.12 |
| References | 65379 |
| DOCUMENT CONTENTS |  |
| Keywords Plus (ID) | 15944 |
| Author's Keywords (DE) | 7123 |
| AUTHORS |  |
| Authors | 12054 |
| Authors of single-authored docs | 38 |
| AUTHORS COLLABORATION |  |
| Single-authored docs | 49 |
| Co-Authors per Doc | 7.02 |
| International co-authorships % | 14.44 |
| DOCUMENT TYPES |  |
| Article  review | 3120  212 |

**Supplementary Table 2.** Most Relevant Countries by Corresponding Author Contributions

| **Rank** | **Country** | **Articles** | **Articles %** | **SCP** | **MCP** | **MCP %** | **TC** | **Average Article Citations** | |
| --- | --- | --- | --- | --- | --- | --- | --- | --- | --- |
| 1 | USA | 289 | 8.7 | 215 | 74 | 25.6 | 14961 | 51.8 |  |
| 2 | CHINA | 206 | 6.2 | 165 | 41 | 19.9 | 4164 | 20.2 |  |
| 3 | ITALY | 128 | 3.8 | 79 | 49 | 38.3 | 4198 | 32.8 |  |
| 4 | KOREA | 127 | 3.8 | 121 | 6 | 4.7 | 3610 | 28.4 |  |
| 5 | GERMANY | 71 | 2.1 | 42 | 29 | 40.8 | 2518 | 35.5 |  |
| 6 | UNITED KINGDOM | 65 | 2 | 37 | 28 | 43.1 | 1723 | 26.5 |  |
| 7 | INDIA | 55 | 1.7 | 45 | 10 | 18.2 | 818 | 14.9 |  |
| 8 | CANADA | 46 | 1.4 | 27 | 19 | 41.3 | 1211 | 26.3 |  |
| 9 | NETHERLANDS | 43 | 1.3 | 27 | 16 | 37.2 | 1686 | 39.2 |  |
| 10 | SPAIN | 39 | 1.2 | 22 | 17 | 43.6 | 1487 | 38.1 |  |

Abbreviation: SCP :Single Country Publications; MCP :Multiple Country Publications; TC: Total Citation

**Supplementary Table 3**. Top 10 Affiliations and Articles in Wearable Device Research Publications for Parkinson's Disease (2005–2026)

| **Affiliation** | **Articles** |
| --- | --- |
| TEL AVIV UNIVERSITY | 123 |
| OREGON HEALTH AND SCIENCE UNIVERSITY | 101 |
| RADBOUD UNIVERSITY NIJMEGEN | 73 |
| UNIVERSITY OF CALIFORNIA SYSTEM | 62 |
| SACKLER FACULTY OF MEDICINE | 60 |
| HARVARD UNIVERSITY | 59 |
| SAPIENZA UNIVERSITY ROME | 50 |
| UNIVERSITY OF ERLANGEN NUREMBERG | 50 |
| EBERHARD KARLS UNIVERSITY OF TUBINGEN | 48 |
| UNIVERSITY OF BELGRADE | 43 |

**Supplementary Table 4**. Original search strategy for wearable and sensor-based monitoring technologies in Parkinson's disease

| Database | Search fields | Original search strategy (standardized English format) |
| --- | --- | --- |
| WoSCC | Title, abstract, and author keywords (TI, AB, AK) | ((TI=("Parkinson's Disease" OR "Parkinson Disease" OR "Idiopathic Parkinson Disease" OR "Idiopathic Parkinson's Disease" OR "Lewy Body Parkinson Disease" OR "Lewy Body Parkinson's Disease" OR "Paralysis Agitans" OR "Parkinson Disease, Idiopathic" OR "Parkinson's Disease, Idiopathic" OR "Parkinson's Disease, Lewy Body" OR "Primary Parkinsonism" OR "Parkinsonism, Primary") OR AB=("Parkinson's Disease" OR "Parkinson Disease" OR "Idiopathic Parkinson Disease" OR "Idiopathic Parkinson's Disease" OR "Lewy Body Parkinson Disease" OR "Lewy Body Parkinson's Disease" OR "Paralysis Agitans" OR "Parkinson Disease, Idiopathic" OR "Parkinson's Disease, Idiopathic" OR "Parkinson's Disease, Lewy Body" OR "Primary Parkinsonism" OR "Parkinsonism, Primary") OR AK=("Parkinson's Disease" OR "Parkinson Disease" OR "Idiopathic Parkinson Disease" OR "Idiopathic Parkinson's Disease" OR "Lewy Body Parkinson Disease" OR "Lewy Body Parkinson's Disease" OR "Paralysis Agitans" OR "Parkinson Disease, Idiopathic" OR "Parkinson's Disease, Idiopathic" OR "Parkinson's Disease, Lewy Body" OR "Primary Parkinsonism" OR "Parkinsonism, Primary")) AND (TI=("Wearable Sensors" OR "Sensors" OR "Biosensing Techniques" OR "Monitoring, Physiologic" OR "Physiologic Monitoring" OR "Remote Sensing Technology" OR "Ambulatory Monitoring" OR "Telemetry" OR "Remote Patient Monitoring" OR "Biosignal Monitoring" OR "Wearable Electronic Devices" OR "Monitoring, Ambulatory") OR AB=("Wearable Sensors" OR "Sensors" OR "Biosensing Techniques" OR "Monitoring, Physiologic" OR "Physiologic Monitoring" OR "Remote Sensing Technology" OR "Ambulatory Monitoring" OR "Telemetry" OR "Remote Patient Monitoring" OR "Biosignal Monitoring" OR "Wearable Electronic Devices" OR "Monitoring, Ambulatory") OR AK=("Wearable Sensors" OR "Sensors" OR "Biosensing Techniques" OR "Monitoring, Physiologic" OR "Physiologic Monitoring" OR "Remote Sensing Technology" OR "Ambulatory Monitoring" OR "Telemetry" OR "Remote Patient Monitoring" OR "Biosignal Monitoring" OR "Wearable Electronic Devices" OR "Monitoring, Ambulatory"))) |
| Scopus | Title, abstract, and author keywords (TITLE-ABS-KEY) | TITLE-ABS-KEY(("Parkinson's Disease" OR "Parkinson Disease" OR "Idiopathic Parkinson Disease" OR "Idiopathic Parkinson's Disease" OR "Lewy Body Parkinson Disease" OR "Lewy Body Parkinson's Disease" OR "Paralysis Agitans" OR "Parkinson Disease, Idiopathic" OR "Parkinson's Disease, Idiopathic" OR "Parkinson's Disease, Lewy Body" OR "Primary Parkinsonism" OR "Parkinsonism, Primary") AND ("Wearable Sensors" OR "Sensors" OR "Biosensing Techniques" OR "Monitoring, Physiologic" OR "Physiologic Monitoring" OR "Remote Sensing Technology" OR "Ambulatory Monitoring" OR "Telemetry" OR "Remote Patient Monitoring" OR "Biosignal Monitoring" OR "Wearable Electronic Devices" OR "Monitoring, Ambulatory")) |

Note: This table presents a standardized English version of the original search concepts used in WoSCC and Scopus. No additional unexecuted terms were added. Search period: 1 January 2005 to 2 February 2026; language: English; document types: articles and reviews.

**Supplementary Table 5.** Benchmark publications used to assess the sensitivity of the final search strategy

| **No.** | **Benchmark publication** | **Year** | **PMID** | **Literature domain represented** | **WoSCC** | **Scopus** |
| --- | --- | --- | --- | --- | --- | --- |
| 1 | Salarian et al. | 2007 | 17278588 | Ambulatory monitoring; tremor and bradykinesia | Yes | Yes |
| 2 | Bächlin et al. | 2010 | 19906597 | Wearable monitoring; freezing of gait | Yes | Yes |
| 3 | Maetzler et al. | 2013 | 24030855 | Quantitative wearable sensors; objective assessment | Yes | Yes |
| 4 | Silva de Lima et al. | 2017 | 28251357 | Wearable sensors; freezing of gait and falls | Yes | Yes |
| 5 | Zhan et al. | 2018 | 29582075 | Smartphone assessment; PD severity estimation | No | No |
| 6 | Powers et al. | 2021 | 33536284 | Smartwatch monitoring; motor fluctuations | Yes | Yes |
| 7 | Oyama et al. | 2023 | 36918552 | Multisensor technology; analytical and clinical validity | Yes | Yes |
| 8 | Adams et al. | 2023 | 37069193 | Smartphone and smartwatch monitoring; early PD | Yes | Yes |

**Note:** The benchmark set was selected purposively to represent major domains within wearable and sensor-based monitoring research in Parkinson's disease. Retrieval was assessed independently in the final WoSCC and Scopus datasets. Seven of eight benchmark publications were retrieved by both databases; one publication (Zhan et al., 2018; PMID: 29582075 ) was not retrieved by either database. The benchmark exercise was used as a practical sensitivity check and was not intended to establish complete retrieval of all relevant literature.
